# Quality improvement in public–private partnerships in low- and middle-income countries: a systematic review

**DOI:** 10.1186/s12913-024-10802-w

**Published:** 2024-03-13

**Authors:** Cassandra B. Iroz, Rohit Ramaswamy, Zulfiqar A. Bhutta, Paul Barach

**Affiliations:** 1 Northwestern University Feinberg School of Medicine, Chicago, IL 60611 USA; 2 James M. Anderson Center for Health Systems Excellence, Cincinnati Children’s Hospital Medical Center, Cincinnati, OH USA; 3 Centre for Global Child Health, The Hospital for Sick Children, Toronto, Canada; 4 Institute for Global Health & Development, The Aga Khan University, South Central Asia, East Africa UK; 5 Thomas Jefferson University, Philadelphia, PA USA; 6 Imperial College, London, UK

**Keywords:** Quality improvement, Public–private partnerships, Low- and middle-income countries, Nutrition, Population health, Implementation, Equity

## Abstract

**Background:**

Public–private partnerships (PPP) are often how health improvement programs are implemented in low-and-middle-income countries (LMICs). We therefore aimed to systematically review the literature about the aim and impacts of quality improvement (QI) approaches in PPP in LMICs.

**Methods:**

We searched SCOPUS and grey literature for studies published before March 2022. One reviewer screened abstracts and full-text studies for inclusion. The study characteristics, setting, design, outcomes, and lessons learned were abstracted using a standard tool and reviewed in detail by a second author.

**Results:**

We identified 9,457 citations, of which 144 met the inclusion criteria and underwent full-text abstraction. We identified five key themes for successful QI projects in LMICs: 1) leadership support and alignment with overarching priorities, 2) local ownership and engagement of frontline teams, 3) shared authentic learning across teams, 4) resilience in managing external challenges, and 5) robust data and data visualization to track progress. We found great heterogeneity in QI tools, study designs, participants, and outcome measures. Most studies had diffuse aims and poor descriptions of the intervention components and their follow-up. Few papers formally reported on actual deployment of private-sector capital, and either provided insufficient information or did not follow the formal PPP model, which involves capital investment for a explicit return on investment. Few studies discussed the response to their findings and the organizational willingness to change.

**Conclusions:**

Many of the same factors that impact the success of QI in healthcare in high-income countries are relevant for PPP in LMICs. Vague descriptions of the structure and financial arrangements of the PPPs, and the roles of public and private entities made it difficult to draw meaningful conclusions about the impacts of the organizational governance on the outcomes of QI programs in LMICs. While we found many articles in the published literature on PPP-funded QI partnerships in LMICs, there is a dire need for research that more clearly describes the intervention details, implementation challenges, contextual factors, leadership and organizational structures. These details are needed to better align incentives to support the kinds of collaboration needed for guiding accountability in advancing global health. More ownership and power needs to be shifted to local leaders and researchers to improve research equity and sustainability.

**Supplementary Information:**

The online version contains supplementary material available at 10.1186/s12913-024-10802-w.

## Background

Large implementation gaps in improving global health and nutrition remain despite abundant funding and research addressing health disparities [1–3]. A recent review found challenges with programming and mixed outcomes for nutritional interventions for children in low- and middle-income countries (LMICs) [4]. Quality improvement (QI), defined as a collaborative effort to improve outcomes, system performance, and professional development [5], is a well-documented approach for improving outcomes worldwide including in LMICs [6]. QI methods can be applied to improve the quality of whole health systems. Recent literature reviews have found that QI can lead to improved outcomes in LMICs including those focused on trauma care [6], surgical infections [7], and antiretroviral treatment [8], although there remains wide variation in individual and system outcomes. We could not find reviews that broadly examined the use of QI aimed at improving health and nutrition in LMICs.

There is a growing interest in understanding how QI can be leveraged in partnerships of international and local non-governmental organizations (NGOs) as well as community groups that function outside of the clinical arena. QI has been successfully applied to community settings to improve population health and primary care, but reviews of literature have been limited to high-income countries [9]. Public–private partnerships (PPP) have become increasingly important to fund and improve health in LMICs and to address the shortcomings of other strategies to improve global health. PPPs in LMICs focus on a variety of topics, including improving population health outcomes. This is because the private sector plays a key part in the delivery of healthcare and public health through procurement of equipment, medicines, ambulances, and technical assistance. However, these are mostly passive. We were interested in the extent to which PPPs are also actively focused on meaningful systems' change and what the literature reveals about how PPPs leverage QI methodologies to achieve their goals. The PPP business model leverages private-sector expertise to improve clinical performance in hospitals and other health facilities and is a useful arena for testing QI impacts [10]. The general perception about PPP is a private sector, for-profit company investing in the government to achieve a particular goal. The prevailing belief is that since the private sector is more efficient and outcome driven, a PPP will be more results-oriented and will hold governments more accountable for meaningful improvements. However, in the LMIC health environments, these kinds of partnerships are relatively few, and most private sector partners are NGOs who are driven by different incentives. In order to begin to understand the use of QI in PPP, we chose a broad definition of PPP to include as many types of PPPs as possible. QI tools and frameworks have been used to improve outcomes for PPP for health and nutrition in LMICs, but little is known about the extent to which these private sector funded approaches are effectively deployed and what are the key factors that contribute to their lasting success [11]. We did not find a systematic overview of this literature [12–16] and found limitations in reviewing the extent and impacts of PPP in middle income countries as well [17].

The objective of this study was to access and richly describe the landscape of QI interventions by PPP in LMICs in non-clinical settings and disciplines (e.g., public health) that influence population health and nutrition. We conducted a systematic literature review assessing the benefits and challenges of PPP-supported QI approaches in LMICs using the PRISMA guidelines. The motivation for the study emerged from a real-world request from an international funder seeking to understand how to best leverage QI methodologies to improve health and nutrition in the contexts of PPPs in LMICs.

## Methods

### Data sources

We searched for English-language studies published before March 2022 using the full text database SCOPUS and grey literature. The search included terms for community and organizations (e.g. community, coalition, population, partner), QI (e.g. quality improvement, continuous improvement, improvement science, plan-do-study-act), health and wellbeing (e.g. health, wellbeing, prevent*), and LMIC (i.e. low- and middle-income countries). The full search strings are available in Appendix A. We used a snowballing literature search technique to manually check the reference lists for additional studies missed in the original database search. Relevant systematic and scoping reviews were reviewed to identify additional relevant articles.

### Study selection

Public–private sector partnerships were defined as programs with a combined deployment of private sector capital and, sometimes, public sector capital to improve public services [18]. Our search examined studies focused on using QI for public health in partnerships aimed at improving population-level public health conditions for social and physical conditions for health and nutrition. Studies exploring the use of QI in hospitals without partnership of public or community groups were deliberately excluded. We chose a broad definition of PPP to capture and assess the various models that are currently described in the literature. One reviewer (CBI) reviewed titles and abstracts to determine if studies retrieved from the search met the inclusion criteria. When the title and abstract did not provide enough information to assess study eligibility, a full-text copy of the study was retrieved and reviewed for inclusion. A detailed discussion with a second reviewer (PB) about the search findings and inclusion study criteria was done with full agreement achieved before proceeding. We did not exclude articles based on study methodology and reviewed all articles including randomized controlled trials, project reports, qualitative interview studies, and editorials. Each study had to meet the following criteria to be included in this review: 1) discuss improvement in one or more LMIC, 2) use a QI approach or methodology, and 3) be conducted within a public–private or community partnership.

### Quality assessment of methods

The methodologic quality of the full-text studies was assessed by one reviewer (CBI) using the Mays and Pope (2000) framework for assessing quality and discussed in great detail with a second reviewer (PB). The methodological quality was assessed based on clarity of the research question, appropriateness of design question, adequate description of the context, robust sampling, systematic data collection and analysis, and reflexivity of the QI account.

### Data extraction

Each article that met the inclusion criteria was abstracted by one reviewer (CBI) using a standardized form, which included key data assessed, study characteristics, setting, design, outcomes, and lessons learned. A second reviewer (PB) reviewed the abstracted data and assessed the overall quality of the data extraction for each selected paper.

### Data synthesis and analysis

We organized the study outcomes in a tabular form including type of PPP, intervention characteristics, outcomes, and direction of effects observed. The interventions were classified based on the components of the intervention that aimed to improve the quality of services. Qualitative data from the selected papers on the study interventions, key roles, discussions, conclusions, and lessons learned were extracted from each article in detail to help guide decisions on real-world observational evidence. We then conducted a thematic analysis, with a phenomenological approach, by first reviewing all the data in detail, then creating initial themes, and finally describing the themes to derive their meaning [19]. Refinement of themes was done through discussion between two reviewers (CBI & PB). The lessons learned were presented in broad themes and widely discussed to better identify the factors for successful PPP interventions, and highlighting the barriers that undermined the effectiveness and/or sustainability of QI programs. This critical appraisal was done to assist decision makers in the identification of high-quality systematic reviews.

## Results

### Search results

**Fig. 1:**
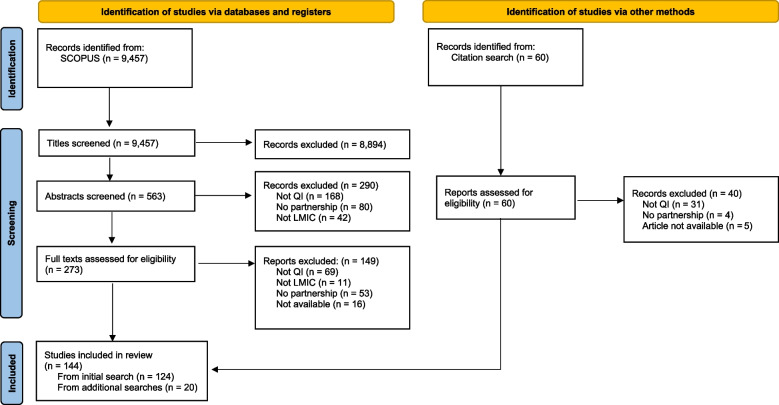
PRISMA flow diagram: summary of evidence search and selection

Our initial search identified 9,457 citations citation (Fig. 1). The title and abstract scan resulted in 273 articles that appeared to meet the inclusion criteria. An additional 60 articles were identified through systematic reviews and references in the included articles. Of these, 20 articles were included in the final analysis. After the full text review, 144 papers met the inclusion criteria and underwent full-text abstraction.

### Characteristics of included studies

**Fig. 2:**
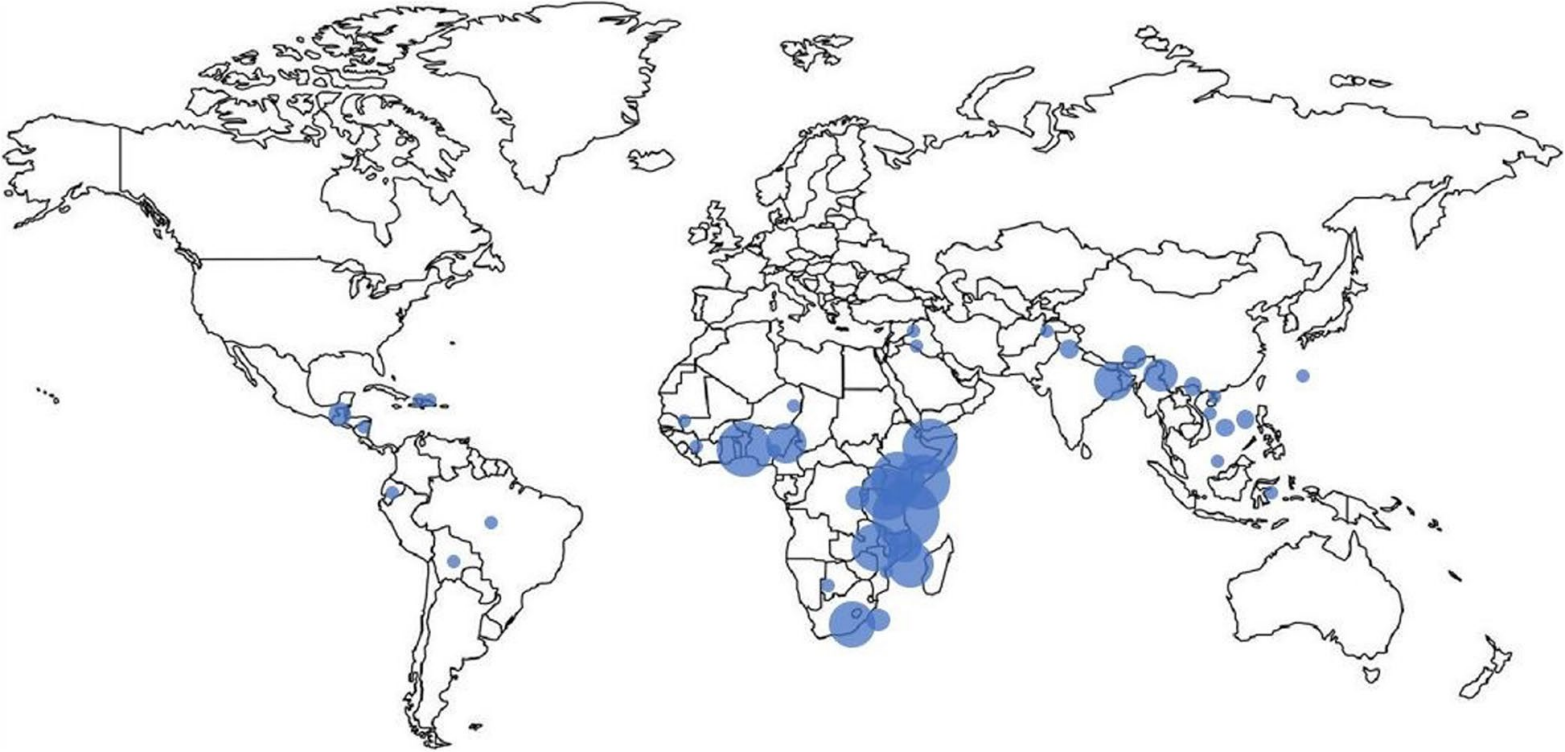
Number of included articles from LMICs across the world. Articles emanated from the following countries: Africa: Burundi (*n* = 1), Côte d'Ivoire (*n* = 1), Democratic Republic of Congo (*n* = 3), Ethiopia (*n* = 16), Ghana (*n* = 17), Kenya (*n* = 17), Lesotho (*n* = 3), Malawi (*n* = 6), Mozambique (*n* = 12), Namibia (*n* = 1), Niger (*n* = 1), Nigeria (*n* = 9), Rwanda (*n* = 15), Senegal (*n* = 1), Sierra Leone (*n* = 1), South Africa (*n* = 12), Tanzania (*n* = 25), Uganda (*n* = 16), Zambia (*n* = 13), Zimbabwe (*n* = 1). Asia: Afghanistan (*n* = 1), Bangladesh (*n* = 6), Cambodia (*n* = 2), India (*n* = 8), Indonesia (*n* = 1), Laos (*n* = 1), Malaysia (*n* = 1), Myanmar (*n* = 2), Nepal (*n* = 3), Pakistan (*n* = 2), Taiwan (*n* = 1), Thailand (*n* = 1), Vietnam (*n* = 2). Latin America: Bolivia (*n* = 1), Brazil (*n* = 1), Dominican Republic (*n* = 1), Ecuador (*n* = 1), Guatemala (*n* = 3), Haiti (*n* = 1), Nicaragua (*n* = 1). Middle East: Jordan (*n* = 1), Lebanon (*n* = 1). The size of the circle corresponds with the number of articles emanating from the given country

Characteristics of the included studies are summarized in Table 1. The studies emanated from projects in 41 countries in Africa, Asia, Latin America, and the Middle East (Fig. 2) with 97 reports from single-country projects, 29 reports from multi-country projects, and 18 reports discussing efforts in LMICs. The main study populations consisted of community health workers, healthcare facilities, healthcare workers, and patients.

**Table 1:** Characteristics of Studies (total 144 articles)

Region^a^	N (%)
Africa	104 (72.2%)
Asia	18 (12.5%)
Latin America	9 (6.3%)
Middle East	1 (0.7%)
LMIC generally	18 (12.5%)
Single Country	97 (67.4%)
Multiple Countries	29 (20.1%)
**Health Focus**^**b**^	**N (%)**
Maternal and Child Health	63 (43.8%)
General Healthcare and Primary Care	41 (28.5%)
HIV/AIDS	25 (17.4%)
Other Infectious Disease	8 (5.6%)
Nutrition	4 (2.8%)
Other	11 (7.7%)
**Partnerships**	**N (%)**
International Governmental and Non-governmental Organizations (NGOs)	104 (72.2%)
Local or National Government and Ministries of Health	80 (55.6%)
Local NGOs	47 (32.6%)
Other Community Partnership	43 (29.9%)
**QI Frameworks and Methods**^**c**^	**N (%)**
Collaborative QI (including IHI Breakthrough Series)	53 (36.8%)
QI without defined framework	38 (26.4%)
Model for Improvement	6 (4.2%)
PDSA Cycles	57 (39.6%)
QI Coaching	29 (20.1%)
Continuous Quality Improvement (CQI)	11 (7.7%)
Monitoring/Quality Assurance	15 (10.4%)
Other	10 (6.9%)
**Research Methods**	**N (%)**
Observational Study	90 (62.5%)
Review or Editorial	26 (18.1%)
Randomized Trial	11 (7.7%)
Qualitative (Interviews & Focus Groups)	11 (7.7%)
Cost-Effectiveness	6 (4.2%)

Africa: Burundi (*n* = 1), Côte d'Ivoire (*n* = 1), Democratic Republic of Congo (*n* = 3), Ethiopia (*n* = 16), Ghana (*n* = 17), Kenya (*n* = 17), Lesotho (*n* = 3), Malawi (*n* = 6), Mozambique (*n* = 12), Namibia (*n* = 1), Niger (*n* = 1), Nigeria (*n* = 9), Rwanda (*n* = 15), Senegal (*n* = 1), Sierra Leone (*n* = 1), South Africa (*n* = 12), Tanzania (*n* = 25), Uganda (*n* = 16), Zambia (*n* = 13), Zimbabwe (*n* = 1)

Asia: Afghanistan (*n* = 1), Bangladesh (*n* = 6), Cambodia (*n* = 2), India (*n* = 8), Indonesia (*n* = 1), Laos (*n* = 1), Malaysia (*n* = 1), Myanmar (*n* = 2), Nepal (*n* = 3), Pakistan (*n* = 2), Taiwan (*n* = 1), Thailand (*n* = 1), Vietnam (*n* = 2)

Latin America: Bolivia (*n* = 1), Brazil (*n* = 1), Dominican Republic (*n* = 1), Ecuador (*n* = 1), Guatemala (*n* = 3), Haiti (*n* = 1), Nicaragua (*n* = 1)

Middle East: Jordan (*n* = 1), Lebanon (*n* = 1)

Maternal and Child: antenatal care, childhood health, childbirth, contraception, family planning, maternal, neonatal, newborn, perinatal, neonatal, obstetrics, and reproductive health

Other Infectious Disease: malaria, tuberculosis, polio, and general immunization

Other: blood transfusion, burn services, chronic obstructive pulmonary disease, critical care, hospital-associated infections, lymphatic filariasis, mental health, palliative care, surgery, supply chain management

*PDSA* Plan-Do-Study-Act, *IHI* Institute for Healthcare Improvement

^a^Percentages do not add to 100% because some studies were across multiple regions (e.g., Africa and Asia)

^b^Percentages do not add to 100% because of some overlap (e.g., maternal HIV)

^c^Percentages do not add to 100% because of some overlap (e.g., PDSA cycles included in model for improvement)

Figure 2 was created for the study. The “Blank Map of the World with Borders” from https://worldmapblank.com/blank-map-of-world/ was used as the background image with the circles representing the number of articles overlayed on top of the map.

The improvement work focused on a variety of health and public health areas. The most common were maternal and child health (*n* = 63), general healthcare and primary care (*n* = 41), human immunodeficiency virus (HIV)/acquired immunodeficiency syndrome (AIDS) (*n* = 25), other infectious diseases (*n* = 7), and nutrition (*n* = 4). Another 11 studies did not fit into one of these categories and included a variety of acute (e.g., burn services) and chronic (e.g., chronic obstructive pulmonary disease) conditions. Some studies were included in multiple categories (e.g., antenatal HIV, childhood nutrition).

The studies were primarily partnerships between funding organizations, government, and private or public healthcare facilities with substantially varying contractual, governance, incentive, and operational structures. The public–private partnerships described intended to influence population health outcomes extending far beyond healthcare settings and represented diverse forms. These PPPs were vaguely described and include private healthcare facilities, healthcare providers, funding (including insurers), and other mentions of “private partnerships” without elaboration. The population level community or public members varied greatly and included international governmental and non-governmental organizations (NGOs) (*n* = 104), local NGOs (*n* = 47), local and national governments and ministries of health (*n* = 80), and community partnerships (*n* = 43). Examples of international partners include Partners in Health, President’s Emergency Plan for AIDS Relief (PEPFAR), The Bill and Melinda Gates Foundation, U.S. Agency for International Development (USAID), Doris Duke Charitable Foundation (DDCF), and UNICEF. The World Health Organization (WHO) was involved in 10 studies and the Institute for Healthcare Improvement (IHI), an international leader in healthcare QI, was involved in 10 studies. We also acknowledge that other types of PPPs may be beyond the scope of this study.

The studies reported on various outcomes including clinical outcomes, processes of care, patient experience, processes of QI, and overall costs (Table 2). Outcomes included patient-, clinic-, and population-levels. We found great heterogeneity in the QI tools, study designs, participants, and outcome measures used. A variety of QI frameworks and tools were used including QI collaboratives (including collaboratives built on the IHI Breakthrough Series Collaborative Model) [20], and the Model for Improvement. Some studies did not reference a clear framework or technique but did refer to QI teams, meetings, and strategies as their intervention. Many studies (*n* = 29) included a QI coach as part of their approach. We included studies that used plan-do-study-act (PDSA) cycles, whether or not they reported using the Model for Improvement.

**Table 2:** Classification of outcome measures from included studies

Outcome Type	Examples of Specific Outcome Measures Used in Studies
Clinical Outcomes	Neonatal mortality HIV infection rate Mother-to-child HIV transmission Adverse drug reaction
Processes of Care	Rates of testing/screening (e.g., tuberculosis, HIV, syphilis) Retention in care (attendance at clinic visits) Number of patients with HIV on retroviral therapy Receipt of antenatal care from a skilled provider Length of stay
Patient Experience	Wait time Patient satisfaction score Patient-reported trust in healthcare team
Processes of QI	Number of QI programs Number of professionals trained in QI Fidelity of implementation of intervention Perceptions of QI (qualitative data)
Cost	Cost of intervention Cost effectiveness

### Methodological quality

We found wide heterogeneity in the reported methods of the included studies. The most common study methodologies were uncontrolled observational studies or descriptive project summaries (*n* = 90), reviews or editorials (*n* = 26), qualitative interview or focus group studies (*n* = 11), randomized controlled trials (RCTs) (*n* = 11), and cost-effectiveness or cost analysis studies (*n* = 6).

### Effectiveness of QI interventions in PPP demonstrated by RCTs

**Fig. 3:**
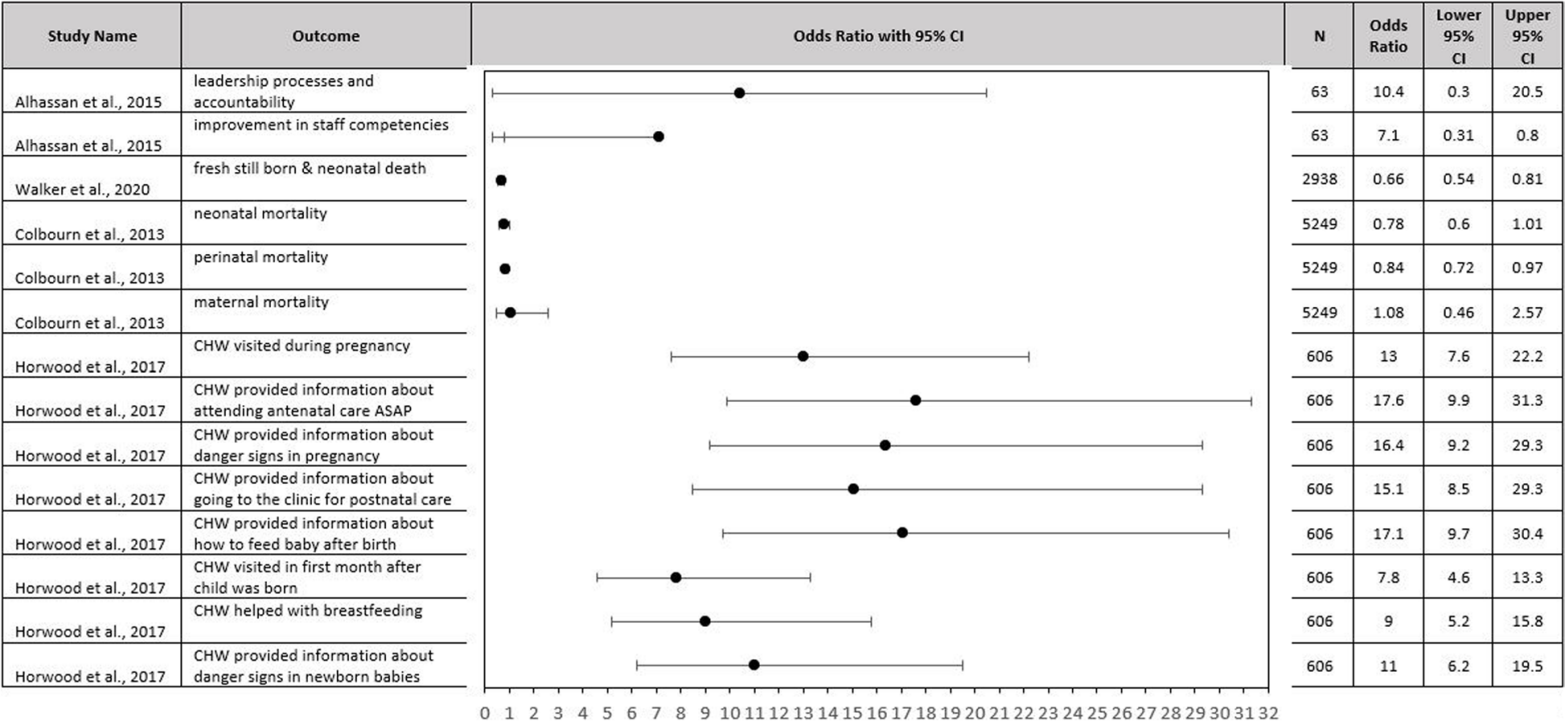
Forest plot of RCTs presenting odds ratios by the study, year of publication, and outcomes. CHW = Community Health Worker; CI = Confidence Interval

Of the 11 RCT reports, only 7 provided data comparing the intervention and control groups. Two reports provided descriptions of the RCT without outcome data [21, 22], one was an interview study of RCT participants [23], and one study provided outcomes only from the intervention group [24]. Four studies provided odds ratios on a total of 14 primary outcomes (Fig. 3). The improvement effect size varied between interventions and between studies. Alhassan et al., found an increase in patient safety efforts in the intervention compared to control facilities [25]. Walker et al., showed a decrease in fresh stillbirth and neonatal mortality for intervention versus control facilities [26]. Horwood et al., showed an increased odds of mothers receiving various types of support from community health workers (CHW) in the intervention arm [27].

The other RCTs showed mixed effectiveness. Colbourn et al., found a statistically significant decrease in neonatal morality and perinatal mortality but no difference in maternal mortality [28]. Osibo et al., found that the time spent accessing services during clinic visits decreased in the intervention arm but found no significant difference in client satisfaction [29]. Oyeledun et al., showed increased rates of early infant HIV testing but no significant difference in retention at 6 months or in initiation of antiretroviral prophylaxis [30]. Finally, Manisha Yapa et al., found that QI significantly increased viral load monitoring but did not improve repeat HIV testing [31].

### Lessons learned from included studies

We identified five overarching themes of key factors leading to effective QI in LMIC partnerships: I) leadership support and alignment with overarching priorities, II) local ownership and engagement of frontline teams, III) shared learning of lessons across teams, IV) resilience in managing external challenges, and V) robust data and data visualization to track progress.

#### Theme I: Leadership support and alignment with overarching priorities

The included studies repeatedly underscored the need for leadership support and alignment with overarching national or organizational priorities to have the resources available for improvement. Many authors discussed how partnerships between government, academia, and communities brought more support and rigor to the studies while maintaining a focus on the needs of the community [13, 32–42]. Having the necessary resources, training, and appropriate knowledge available for QI was discussed as an important aspect of the teams’ willingness to change [29, 43, 44]. Working with governments and Ministries of Health and leveraging existing infrastructure was specifically discussed as essential for leadership engagement and sustainment of improvement efforts [44–55]. Other studies described challenges when they lacked the appropriate support or policy to sustain their improvement efforts [56–61]. Government-run health systems are common in LMICs, requiring more governmental support for effective use of QI across the system [62]. An additional benefit of aligning QI with ongoing strategic priorities was that it reduced the additional workload on frontline staff [43, 63–67]. While unchecked and unaccountable power by executives without oversight can be problematic, studies in our search showed that projects had more resources when they had explicit leadership support that was unambiguous. Projects struggled to achieve their goals when the support was lukewarm, inconsistent, and misaligned to the vision of executive leadership.

While alignment with priorities was a strength, it was also identified as a challenge in assessing the true impacts of the QI approach. For example, one study discussed how it was difficult to determine if improvement was due to their work, or from other contemporaneous maternal, newborn, and child health program interventions in the region [68]. Another study discussed how other HIV/AIDS interventions might have improved care in the control group, blunting the ability to infer change by the QI intervention group [30].

#### Theme II: Local ownership and engagement of frontline teams

One of the primary benefits of using QI approaches was the focus on empowering the frontline teams to champion the improvement efforts. Authors of the included studies described the importance of engaging frontline staff, who understand the context and culture of the community, as well as the unique contextual problems and solutions that were likely to be acceptable and sustainable [26, 34, 45, 58, 69–81]. Many authors described a need for culturally specific interventions in LMIC settings [82–85]. Local ownership and interventions that were tailored to the local culture were important for individuals’ willingness to change [86]. Others expressed that QI methods allowed frontline teams to adapt interventions to their local contexts [13, 15, 49, 74, 87–95].

Strong community engagement was discussed as a core component of many of the projects [35, 54, 77, 85, 91, 96–101], including the development of partnerships with local organizations, not just international funders [102]. Teams were able through local engagement to leverage existing local resources, which aided in conducting and sustaining projects in resource-constrained settings [24–26, 31, 48, 59, 61, 89, 103, 104]. A common theme across QI projects was the community and frontline staff ownership of the system improvements [23, 36, 44, 49, 74, 86, 105–111], which often led to a high uptake of interventions [29, 112, 113], and sustainable changes [75, 114, 115]. For example, in one program in Zimbabwe aimed at eradicating malaria, the program focused specifically on empowering frontline workers to take ownership, solve problems, and act on decisions, and deliberately worked to increase ownership and accountability by conducting team building, awarding best performing districts, and providing peer support visits [34]. In contrast, another program in Ethiopia discussed how the QI teams were poorly integrated within the urban health extension program, leading to a lack of ownership of the QI initiative and a perception that the community-based intervention was an additional burden imposed on the system by the health center staff [61]. Others discussed how QI improvements led by the community and frontline staff were more effective in contrast to external audits which were viewed as punitive [55]. Teams were able to build trusting relationships [52, 56, 111, 116] by working closely with communities to implement QI efforts and focusing on problem-solving rather than fault-finding [12, 117–119].

#### Theme III: Shared learning of lessons across teams

The ongoing sharing of meaningful data and authentic lessons learned were described as common benefits of collaborative QI programs. Several of the included studies used a QI collaborative framework, such as the IHI Breakthrough Series model, which allowed for teams to talk with other teams working on QI projects. Team members valued the opportunities for peer-to-peer learning and the ability to learn directly from others working on similar projects in different settings [34, 36, 44, 54, 76, 82, 89, 95, 108, 111, 119–122]. QI tools could be applied to a wide variety of disciplines, and the teams benefitted from building QI capacity and learning techniques rather than focusing solely on one improvement project [36, 47, 69, 77, 94, 99, 122–124]. Many authors described the benefits of having coaches or external experts to teach QI methods, provide mentoring support, and motivate perseverance in the face of obstacles [51, 58, 63, 92, 93, 124].

Many authors discussed how QI work is often not published, which limits the ability to accurately assess their impact and share learnings across programs and geographic regions [13, 15]. The authors emphasized the importance of sharing across sectors and regions as community partners working in LMICs could benefit from shared learnings including on what intervenions have worked and what have failed.

#### Theme IV: Resilience in managing external challenges

Many of the authors discussed the challenges they faced and the resilience that was needed to overcome organizational, cultural, and resource constraints. Barriers to effective QI programs are common in virtually all settings, but especially in LMICs [28] given limited resources [12, 13, 40, 45, 56, 58, 66, 77, 84, 95, 96, 122], competing priorities [51, 94], natural disasters [91], ambiguous governance [12, 13], and political and social strife [29, 30, 44, 46, 75, 77, 105]. A common challenge noted in the included studies was high turnover of nurses, healthcare staff, and QI team members, which made ongoing leadership directions and sustained changes difficult [31, 37, 45, 47, 51, 53, 55, 81, 94, 122, 125] and led to loss of organizational memory and trust [15]. Others noted that QI efforts might take more time in a community setting than in higher-resource healthcare settings, which should be considered when funding and planning for LMIC QI interventions [126].

While resources were limited, QI could also be cost-effective. All six studies evaluating the costs of QI programs in partnerships in LMICs found the QI approach to be cost-effective [24, 56, 123, 127–131]. One author noted how local NGOs can compete for funding and resources but collaborative QI had the opportunity to bring them together to meaningfully collaborate and learn from each other and save resources [38]. Additionally, funders have an opportunity to incentivize and reward QI lessons [132]. Despite the cost-effective nature of these strategies, there is still a pervasive financial commitment needed from external donors to address the pervasive sustainability challenges [39, 46, 59, 75, 80, 83, 100, 104, 133–135]. Startup activities are especially resource intensive [69], but QI-based approaches appear to be cost-effective in the long run.

#### Theme V: Robust data and data visualization

The use of continuous, reliable, transparent data, and tools for data visualization emerged as a key theme for successful improvement efforts in LMIC settings. Many authors discussed the need for data to continuously measure progress of improvement efforts [26, 52, 55, 81, 82, 100, 101, 111, 119, 132]. Real-time monitoring of data and feedback allowed for continuous and rapid improvements [45, 64, 72, 73, 85, 90, 95, 96, 113, 122, 136]. Run charts were one common tool used for data visualization, and teams felt motivated by seeing their improvements displayed using a run chart [49, 63, 92]. Data dashboards also helped identify bottlenecks, set priorities, and focus QI leadership attention when a change was needed [12, 36, 87, 93, 120]. Collecting and analyzing data was also described as important for studying a pilot version of an improvement project before scaling up [33, 68, 137].

Some authors discussed the benefits of national data monitoring systems to systematically collect data and allow for meaningful comparisons across differing contexts [36, 138], and to increase the uptake of results [35, 49, 55, 64]. Many authors noted that data must be relevant to the local context as QI indicators in LMICs can often differ from those in high-income settings [13]. Some authors discussed the need to develop better quality indicators [34], or look beyond traditional metrics to identify data that are important and make sense in the local context (sense-making) [73, 139, 140].

There were additional challenges regarding data interpretation and effective data visualization. Many programs were challenged with capacity and resources to collect and analyze data [13, 35, 93, 126, 141]. There were concerns about the reliability of data and a need to create better data collection systems [15, 37, 44, 55, 77, 81, 88, 94]. Some authors discussed the urgent need for better measurement and data analyses that required minimal technical support and leveraged existing resources [24, 25, 59, 66, 67, 108, 119, 132, 133, 137]. Others reported that it was beneficial to integrate the QI data collection into existing workflows rather than creating additional tasks for already overworked teams [64, 65, 67]. Building upon existing data systems was essential for building sustainable capacity [26]. Another challenge with data analysis was information sharing, due to various governance constraints related to data privacy, data sharing limits, and regulatory roadblocks [42, 121, 142]. We noted yet another challenge in which project sites used different methodologies for collecting data, making cross-comparisons and meaningful and reliable learning difficult [108, 121].

## Discussion

Our systematic literature review found that most QI interventions in LMICs were multi-component and most studies faced many of the same challenges that QI projects must address in high-income countries. This is the first study to examine the current literature on the use of QI in public–private and community partnerships in LMICs.

The articles included in our review reported the need for robust leadership, frontline engagement, ongoing learning, efficient resource management, and systems for data collection. Leadership support is needed to build infrastructure to facilitate QI, and for alignment with governments and Ministries of Health, especially important in low-resource settings. Engagement of frontline workers, community members, and local NGOs creates interventions that are tailored to the unique needs of the community, increasing acceptability and sustainability. Sharing across sites is valued and can be facilitated by international organizations. Resource limitation is a common challenge for LMICs, and partnerships with external organizations can provide resources, funding, and QI expertise to support local teams. Tools to visually manage data are essential to motivate systemic change so that data collection and application to QI efforts becomes the normal default. We note that several studies included in our review discussed national monitoring systems as important facilitators to drive QI efforts. However, while access to data is necessary, it is insufficient to motivate sustainable changes without systemic support.

The themes we drew from the included articles are similar to the themes that have been demonstrated in other, non-PPP settings. A review of a national program in the United States described the interactive elements critical to successful transformation: impetus to transform, leadership commitment to quality, improvement initiatives that actively engage staff in meaningful problem solving, alignment to achieve consistency of organization goals with resource allocation, and integration to bridge traditional intra-organizational boundaries [143]. Previous literature reviews have found that leadership, organizational culture, data infrastructure, microsystem motivation to change, and abundant resources are important for QI success [144]. Additionally, it is already widely accepted that factors in the organization and external environment in healthcare can have a large impact on the ability of QI teams to accomplish their goals [145]. The themes that emerged from the studies included in this review are therefore not surprising.

While we identified many themes on lessons learned, the articles included in our review rarely provided enough strategic and tactical data to understand how these concepts were operationalized and the organizational and resource constraints. This limited our ability to provide concrete examples of best practices for QI in public–private partnerships in LMICs. The lack of robust descriptions of the QI initiatives, poor methodologic quality, and a limited use of implementation frameworks make it difficult to understand the contextual factors that guide lasting success. For example, while ownership and engagement of frontline team members in the QI work was described as essential for QI success, the descriptions of how frontline staff were engaged in leading efforts were rarely provided. Additionally, while the teams valued the ability to share and learn from one another in QI collaboratives, the details of how learning lessons happened under real-world conditions and how those lessons were shared were rarely reported. The studies mostly lacked discussion of the participants’ competencies and the programs' willingness and culture to change.

We aimed to understand how QI has been applied in LMIC settings, however, we were limited by the poor descriptions of the QI programs and implementation details. There was very little substantive content on approaches to build systems of quality measurement, and motivate workers to routinize sharing these measures, appealing to intrinsic and extrinsic motivation for change. Although many of the included articles use well-known and highly studied approaches, many either did not use a formal QI framework from the literature or did not reference it in their published articles. There was no strong evidence that a single intervention was associated with positive effects on a specific outcome measure. Most multicomponent QI interventions were vaguely reported, making it difficult to determine the fidelity with which they were applied. In the few RCTs that we identified, the results were largely positive (Fig. 3). However, there was a lack of high-quality RCTs and publication bias might lead to negative results not being shared [146].

We also aimed to understand the role of public–private and community partnerships in LMICs and their impacts on population health. However, we were limited by the vague definitions and descriptions of the PPPs, with few papers formally screening for actual deployment of private-sector capital. The authors typically reported that the project was a partnership between public, private, and community organizations, listing the names of the organizations, but not providing information on how the partnership was governed, how the capital was used, and its impact on the project’s effectiveness. We also found limited discussion on the role of private-sector partners. These partnerships do not follow the traditional PPP model, which is a formal partnership with capital investment for a return on investment. The definition of PPPs appears to be misused in the literature and the descriptions of private sector, for-profit company investing in the government to achieve a particular goal are extremely limited. Instead, we found that almost all the reported results were partnerships between governments and private sector NGOs, who are driven by different incentives. Because of this we were unable to study other types of partnerships. We chose to define PPPs broadly to be able to assess the differences between types of PPPs, but due to the partnerships described in the literature we were ultimately unable to do so, and instead we reported the results as an aggregate. While rare, there were some descriptions of true PPPs, such as the one between the government of Lesotho and a private consortium of hospital services [10]. In this example, the important drivers of success included better defined policies and procedures, empowerment and training of managers and staff, and increased accountability, as supported by changes in infrastructure, communication, human resource management, and organizational culture [147].

In addition to the issue that the PPPs described in the literature do not meet the formal definition of for-profit companies investing in the government efforts, the details on the structure of the partnerships included was unavailable. Discussion of roles, responsibilities, shared vision, common goals, trust, and respect are needed to understand these relationships and their potential impacts on implementation success. This lack of detail makes it difficult to understand the governance and leadership structures overseeing the QI programs and what type of governance structures are most effective. We did not find evidence that any one governance structure better supports effective QI projects in LMICs.

The QI lessons learned emerged primarily from the discussion and conclusion sections of the reviewed articles. While these reflections by the authors added valuable insights into their perspectives on what led to success, the barriers impeding improvement, and lessons learned for future initiatives, they are limited in their inferential ability to offer a full and robust understanding of the key factors causally contributing to the reported outcomes. The vague descriptions of leadership, culture, and roles greatly undermined our ability to learn from these studies. We, and others, recognize the opportunity to systematically use implementation science to better appreciate the organizational and cultural contextual factors and determinants of success in QI initiatives in these challenging settings [16, 73, 113, 148]. Further empirical research using qualitative and quantitative approaches would be helpful to determine the full extent of the implementation challenges including the context, organizational culture, and user involvement.

The literature reviewed demonstrates that many QI programs have been able to improve health outcomes in LMICs through the use of QI frameworks. The randomized trials, while limited, demonstrated positive or mixed effectiveness. Promising interventions for improving PPP QI efforts in LMICs exist but require further investigation. These projects and their evaluations support our convictions on the need to prioritize authentic co-design of QI efforts, led by local stakeholders through coaching, to drive effective PPP-funded QI implementation efforts. Local leaders have a greater understanding of the local barriers blocking QI uptake and the enabling factors for sustainable success. Additionally, more studies on cost-effectiveness of PPPbased QI efforts are needed as resource limitations are a huge and ongoing issue for LMIC partnerships and their lasting impacts.

### Research equity

In discussing partnerships between international organizations and LMICs, it is essential to consider the issues of equity, vision, and goals and how these factors can influence how a partnership is planned, implemented, and reported. There has been a recent movement in partnerships with LMICs to shift the agency, leadership, power, and ownership to local researchers and community leaders [149]. Researchers in LMICs have described the challenges they face that are often not understood by high-income country funders and researchers, and have called for a more active and meaningful role in decision-making, research planning, and study implementation [150]. These power imbalances are increasingly seen as a major factor in the underperformance of PPP-directed QI projects in LMICs and have been thrust to the forefront during the COVID-19 pandemic [151]. Some articles in our review discussed a need to focus on equity in QI planning, staff engagement and sustainable work [73]. One study by Muller et al. specifically noted how the “quality movement was driven by the United States” [152] as opposed to the need for locally derived solutions that are sensitive to local contexts and are meaningful to local stakeholders.

## Limitations

Our study has several limitations. First, due to the broad range of methodologies, we were not able to report on each of the approaches in depth. Second, many of the studies had diffuse aims and lacked sufficient data and clear descriptions of the programs, limiting the ability to meaningfully assess what interventions led to the reported outcomes. Third, the populations varied greatly, limiting the ability to effectively compare interventions across studies. Fourth, our review deals with complex interventions, including a number of interactions between components, variability of outcomes, and the permitted degree of flexibility or tailoring of the interventions [153]. These aspects can hinder an appropriate and direct evaluation of the interventions. Fifth, our study was limited to English-language studies, potentially overlooking reports written in other languages. Sixth, while we aimed to review the literature on population level public health conditions by using search terms for health, wellbeing, and nutrition, public health is a broad field that creates the social and physical conditions in which people can be healthy [154]. Our review does not encompass all of the structural factors of public health (such as education, sanitation, housing). Additional work examining the use of QI for all aspects of physical and organizational infrastructure and capacity building in communities would be interesting and beyond the scope of this already expansive review. Seventh, many of the included studies either did not provide enough information to understand if or how private-sector capital is deployed or did not follow the formal PPP model, which is a formal partnership with capital investment for a return on investment. Eighth, PPPs exist outside of population health and we did not examine the financial structure of PPPs that leverage QI in other sectors, beyond the scope of this study, that might have valuable, transferrable lessons learned. Ninth, screening for eligibility and data extraction were performed by a single reviewer although all study findings were carefully reviewed, and all conclusions agreed to by the second reviewer. Finally, our review may be influenced by publication bias [146]; while we made an effort to search grey literature to gain a thorough breadth of QI work in LMICs, there is likely much more work that has not been published in peer-reviewed literature or available in the sources we explored, or due to unpublished negative results.

## Conclusions

Many interventions aimed at improving QI in LMICs have made marginal impacts on global health through PPPs. The factors that emerged as important (including a clear vision, robust leadership support, frontline engagement, ongoing learning, and managing resources efficiently) were not surprising as these are well studied in other settings. Our study indicated that despite the promise of PPPs, we are currently unable to fully assess whether PPPs are in any way different or better from other funding organizations in LMIC improvement efforts. The descriptions of the interventions' aims and components, heterogeneity of the interventions and study characteristics, and validity of the outcome measurements, hinder the demonstration of robust evidence in supporting the effectiveness of the interventions. The papers we found in our exhaustive literature search say too little about how QI is actually implemented in PPPs, which makes it difficult to understand how to improve private sector funded QI implementation in LMICs. Most of the PPPs we studied were partnerships between NGOs and governmental entities where there is little incentive for accountability. Additionally, most of the programs were focused on project processes rather than outcomes. Third, we found that implementation of QI is unsystematic and poorly documented.

There is an ongoing need to understand how PPPs could potentially improve QI outcomes, how NGO/governmental partnerships support or impede this, and the implications for future research. Future research should develop a clearer description of the PPP funding and incentives structures, QI interventions, use uniform and valid outcome measures, appropriate study designs for assessing the impact and implementation, and attend to the local LMIC stakeholders' needs in developing effective QI interventions. A focus on implementation science is needed to better understand the organizational and cultural contextual factors that lead to successful improvement, and to inform alignment of the kinds of collaboration needed for achieving global health outcomes.

### Supplementary Information

**Supplementary Material 1.**

## Data Availability

All data are from currently published studies cited in the paper. Data summarized used for during the current study are available from the corresponding author on reasonable request.

## Abbreviations

QI: Quality improvement
PPP: Public-private partnership
LMIC: Low-and-middle income countries
NGO: Non-governmental organization
HIV: Human immunodeficiency virus
AIDS: Acquired immunodeficiency syndrome
IHI: Institute for Healthcare Improvement
PDSA: Plan-do-study-act
RCT: Randomized controlled trial
CQI: Continuous quality improvement
CHW: Community health workers
