# Supplementary material for: Quality improvement in public–private partnerships in low- and middle-income countries: a systematic review
Source: BMC Health Serv Res. 2024 Mar 13;24:332. doi: 10.1186/s12913-024-10802-w (PMC10935959; doi:10.1186/s12913-024-10802-w)
Supplement: Supplementary file 1 — Supplementary Material 1. [file 12913_2024_10802_MOESM1_ESM.docx]

**Appendix A. Detailed listing of search terms**

( TITLE-ABS-KEY ( "Health department" OR ( community AND coalition* ) OR ( community AND partner* ) OR communit* OR ( community AND organization* ) OR population* OR ( low AND middle AND income ) ) AND TITLE-ABS-KEY ( "Quality improvement" OR "improvement science" OR "continuous improvement" OR "improvement methods" OR "improvement tool" OR "quality initiative" OR "quality tool" OR "quality methods" OR "driver diagram*" OR "value stream mapping" OR "model for improvement" OR "process improvement" OR ( lean AND ( quality OR management ) ) OR "six sigma" OR "lean six sigma" OR pdsa OR "plan do study act" OR "plan-do-study-act" OR "plan do check act" OR "plan-do-check-act" OR pdca ) AND TITLE-ABS-KEY ( health* OR wellbeing OR well-being OR well-being OR "well being" OR prevent* OR academic* OR education* OR justice OR "social determinant" OR equit* OR housing ) AND NOT TITLE-ABS-KEY ( genetic OR biopsy OR surgery OR "long-term care" OR "nursing homes" OR perioperative OR postoperative OR radiology OR "patient safe-ty" OR "patient protection" OR "patient care" OR "patient discharge" OR "length of stay" OR "medical errors" OR "intensive care unit" OR "patient-reported outcomes" OR pros OR "enhanced recovery path-ways" OR erps OR "medical education" OR "postgraduate training" OR "clinical clerkship" OR "hospital administration" OR microbiome OR gene OR engineering ) )
